# Biological Evaluation of the Isolated Compounds from Methanol Fraction of* Leutea avicennia* Mozaff

**Published:** 2018

**Authors:** Mahsa Sabernavaei, Farzad Kobarfard, Abbas Hadjiakhoondi, Majid Aghaahmadi, Mohsen Amin, Narguess Yassa

**Affiliations:** a *Department of Pharmacognosy, Medicinal Plants Research Center, Faculty of Pharmacy, Tehran University of Medical Sciences, Tehran, Iran.*; b *Depatrtment of medicinal chemistry, School of Pharmacy, Shahid Beheshti University of Medical Sciences, Tehran, Iran.*; c *Department of Botany, University of Isfahan, Isfahan, Iran.*; d *Depatrtmentof Drug and Food Control, and Pharmaceutical Quality Assurance Research Center, Faculty of Pharmacy, Tehran University of Medical Sciences, Tehran, Iran.*

**Keywords:** Leutea avicennia, Apiaceae, AChE, DPPH, Phenolic compounds

## Abstract

*Leutea avicennia* Mozaff. That belongs to Apiaceae family is an endemic species distributed in the west of Iran. The aim of this study was to investigate the antioxidant activity and acetylcholinestrase (AChE) inhibition of the crude extract, fractions, and isolated compounds from methanol fraction of *L. avicenniae.* Five compounds were detected from methanol fraction; three phenolic compounds as p-coumric acid, caffeic acid, salicylic acid and also, two flavonoids as quercetin and astragalin. These structures were identified by spectroscopic techniques such as ^1^H-NMR, ^13^C-NMR, and UV. Antioxidant activity was evaluated by the free radical scavenging assay using 2, 2-diphenyl-1-picryl- hydrazyl (DPPH) method. Ellman colorimetric method was used to determine acetylcholinestrase (AChE) inhibition. In the DPPH assay, Quercetin exerted the highest antioxidant activity (IC_50 _= 10.24 ± 1.3 µg/mL). Caffeic acid inhibited AChE with IC_50 _= 12.06 ± 2.01µg/mL which were comparable to Galanthamine as positive control (IC_50 _= 62.44 ± 2.2µg/mL). In conclusion, methanol extract of* L. avicenniae* contains bioactive components with antioxidant and AChE inhibitory effects.

## Introduction

Free radicals, along with oxidative stress reactions can lead to damage biological molecules like DNA, proteins, and lipids (1, 2). Therefore, oxidative stress has been involved in pathogenesis of various diseases including cancers, atherosclerosis, and neurodegenerative disorders such as Alzheimer’disease (AD) (3, 4). Pathophysiology of AD is relevant to oxidative stress and evacuation of neurotransmitter acetylcholine by acetylcholinesterases (5). A prevalent method for treating AD is to increase the ACh level in the brain utilization AChE inhibitors (6). The chemical medicines have limitations associated with side-effects. The search for discovering strong new AChE inhibitors from natural origins without side effects is great attention (7). Currently, a number of treatments are applied against Alzheimer’s disease too to answer the effect of oxidative stress. These consist of the use of acetylcholinesterase inhibitors (AChEIs) and large amount of antioxidant (5). 

Plants supply a valuable resource, functional as leads to the extension of therapeutic compounds (8). Regular consumption of numerous plant species as daily diet and use of natural health products (NHPs) as medication urged the scientists to conduct extensive research on the antioxidant and AChE inhibitory effects of phenolic compounds in the treatment of AD (9).

The genus *Leutea* is represented by 11 taxa widespread throughout central Asia and Iran (10). Recently, this genus has been categorized as *Ferula* group (*Ferula*, *Dorema*, and *Leutea*) (11, 12). Since* Dorema* and *Ferula *genera have strong antioxidant activity, this study was conducted on* Leutea avicenniae* for the first time (12, 13).

The extracts and diastereoisomers of *Ferula lutea *were examined for antioxidant activity and AChE inhibitory effects. The ethyl acetate extract had the strongest antioxidant activity (IC_50 _= 12.8 ± 1.29 µg/mL) (13). Another investigation reported antioxidant and anti-hemolytic activities of *Ferula gummosa* Boiss.and its extract exerted moderate antioxidant activity (IC_50 _= 579.6 ± 19.4 µg/mL) which was comparable with vitamin C (14).

In a study, antioxidant effects and AChE inhibitory effects of quercetin and astragalin isolated from *Gossypium herbaceum* were examined showing strong antioxidant effects (IC_50_=0.84 µg/mL) and acetylcholinesterase inhibitory (IC_50 _= 50.99 µg/mL) (15).

In this study, we report the results of the antioxidant and AChE inhibitory effects of* L. avicennia* extract and it’s identified compounds isolated from the methanol fraction.

## Experimental

*Chemicals*

Acetylcholinesterase (AChE) and Acetylthiocholin iodide (ATCI) was purchased from Sigma (Germany). 5, 5′-dithiobis-(2-nitrobenzoic acid) (DTNB) and other chemicals and solvents were provided from Merck (Germany).

*Plant material*

Aerial parts of *L. avicennia* were collected during flowering period from Kaboudarahang Mountain (2200 m) in Hamadan Province (West Iran), in August 2012 and identified by botanist Majid Aghaahmadi. The voucher specimen of the plant (Voucher No. 6761-TEH) was kept in the Herbarium of the Faculty of Pharmacy, Tehran University of Medical Sciences, Tehran, Iran.

*Methods*

*Extraction and fractionation*

700g of the air dried aerial parts of plant was macerated with 80% methanol (4 L ×5) at room temperature and was concentrated with an evaporator in low temperature (40 °C). The crude extract (160 g) was sequentially fractionated with petroleum ether, chloroform, ethyl acetate, and methanol (each 4 L) respectively, to obtain four fractions.

*Acetylcholinestrase (AChE) inhibitory activity of Fractions*

AChE inhibitory effect of petroleum ether, chloroform, ethyl acetate, and methanol fractions were carried out by the Ellman′s method (16). Galantamine was used as a positive control and distilled water as negative control.

In this method, 100 µL of 0.1 mM sodium phosphate buffer (pH = 8.0), 20 µL DTNB (5,5′-Dithiobis (2-nitrobenzoic acid) 20 µL of sample solution, 2 µL of AChE solution were added into 96 micro plates and were incubated for 15 min at 25 °C and then acetylcholine iodide (100 µL of 0.05 mM water solution) was added as substrate. Acetylcholinestrase activity was evaluated by measuring the absorbance at 412 nm by an ELISA plate reader for 3.0 min at 25 °C. The concentration of the compound which inhibited 50% of acetylcholinestrase activity (IC_50_) was calculated (Table 1). 

**Table 1:** Antioxidant Activity and AChE Inhibitory effects of extract, fractions and isolated compounds of *L.* *avicennia*

**Samples**	**Antioxidant Activity IC**_50_ ** ± SD**	**AChE Inhibitory IC**_50_ ** ± SD** *
Crude extract	125.20±1.0	-
Petroleum ether fraction	253.40±2.2	-
Chloroform fraction	106.70±4.8	-
Ethyl acetate fraction	85.40±4.3	-
Methanol fraction	49.80±7.0	205.10±3.3
Quercetin	10.24±1.3	66.50±2.4
Astragalin	26.92±3.5	72.80±1.8
Caffeic acid	11.41±6.0	12.06±2.0
Salicylic acid	114.57±1.0	-
p-Coumaric acid	110.33±1.2	-
BHT	19.50±2.8	ND
Galantamine	ND	6.24±2.2

ND: not determined;

* : µg/mL; - not active

*DPPH radical scavenging activity*

The DPPH method is based on the free radical scavenging activity (17). briefly, 2 mL of DPPH solution in methanol (4×10^-5^ g/mL) was prepared and added to 1 mL of different concentrations of sample solution in methanol (0.1, 0.5 and 1.0 mg/mL). The mixture was kept in the dark for 30 min at room temperature. The antioxidant activity was measured by Shimadzu, UV/VIS model 160A spectrophotometer at 517 nm.

All the measurements were done in triplicate. The IC_50_ values were calculated as means ± SD and butylated hydroxytoluene (BHT) was used as positive control.

*Isolation and identification of compounds*

The potent methanol fraction (20 g) was subjected to Sephadex LH-20 (Fluka, Switzerland) column chromatography using 100% methanol as eluent. This was followed by increasing water up to 50% resulting in 3 fractions (M_1_-M_2_-M_3_). Fraction M_1_ (1.5g) was re-chromatographed over a Sephadex LH-20 and eluted with MeOH-H_2_O (6:4) to collect compound **1** (8 mg). Fraction M_2 _(700 mg) was run on a C_18 _reversed-phase (230-400 mesh, fully end capped, Fluka, Switzerland) column chromatography using MeOH-H_2_O (8:2) as eluent, and compound 2 (11 mg) was achieved.

**Figure 1:**
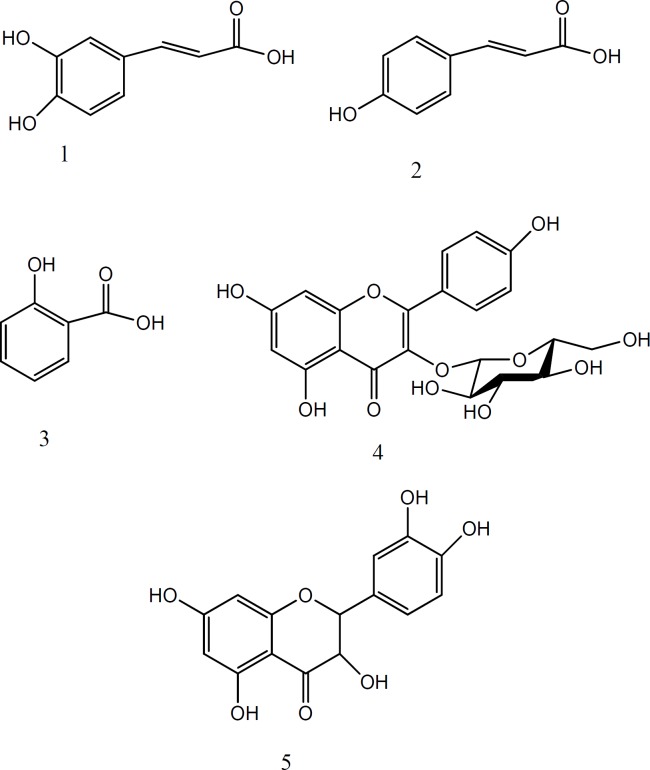
Structure of the isolated compounds, 1- Caffeic acid, 2- p-Coumaric acid, 3- Salicylic acid, 4- Astragalin, 5- Quercetin

Compounds 3, 4, 5 were isolated from M_3_ fraction using paper chromatography (PC, Whatman No.1) and solvent system BAW (butanol: acetic acid: water, 4:1:5). For further purification the subfractions were subjected to PC on Whatman No.1 with 15% acetic acid, then, they were re-chromatographed over sephadex LH-20 to earn compound 3 (12 mg, R_f _= 0.4, blue before and after using NH_3 _vapor at 366 nm), compound 4 (9 mg, R_f _= 0.59, yellow before and after using NH_3 _under 366 nm), and compound **5** (250 mg, R_f _= 0.34, dark blue before and after using NH_3 _under 366 nm) (18, 19).The isolated compounds were identified with different spectroscopic methods (^1^H-NMR, ^13^C-NMR and UV) as quercetin, astragalin, caffeic acid, salicylic acid and p-coumric acid (Figure1).

*Acetylcholinestrase inhibitory and antioxidant activity of isolated compounds*

AChE inhibitory and antioxidant effects of isolated compounds: quercetin, astragalin, caffeic acid, p-coumaric acid and salicylic acid were examined by the Ellman′s and DPPH methods respectively for determination of effective compounds (Table 1).

## Results and Discussion

*L. avicennia* is an endemic plant of Iran and the purpose of this study was evaluation of antioxidant and AchE inhibitory effects of the plant extract, fractions, and isolated compounds. According to the antioxidant test, methanol fraction exerted strong free radical scavenging activity (IC_50 _= 49.8 ± 7.0 µg/mL) compared to the other fractions. Quercetin exhibited the highest free radical scavenging activity with IC_50 _= 10.24 ± 1.3 µg/mL compared to BHT as positive control (IC_50_=19.5 ± 2.8 µg/mL) while the lowest activity belonged to salicylic acid (IC_50_=114.57 ± 1.0 µg/mL). Regarding the inhibition of acethylcholinestrase, methanol fraction showed inhibitory activity (205.10 ± 3.3 µg/mL) among other fraction and caffeic acid (IC_50_ = 12.06 ± 2.01 µg/mL) was stronger than other isolated compounds.

The antioxidant activity and AChE inhibitory assay of the total extract, fractions, and compounds are listed in the Table 1.

Isolation and purification of compounds from methanol fraction, has been done by various chromatographic techniques, including PC, and column chromatography (LH 20, normal and reversed–phase silicagel). Finally, five compounds were isolated and identified with different spectroscopic methods (^1^H- NMR, ^13^C-NMR, and UV) and were compared with the reported data, including quercetin (20, 21, 22), astragalin (20, 23), caffeic acid (24, 25), salicylic acid (26) and p-coumric acid (27, 28, 29) (Figure1). 

In previous study, quercetin(IC_50_=19.8 µg/mL). and 3-methoxy quercetin (IC_50_=37.9 µg/mL) isolated from *Agrimonia* had remarkable AChE inhibitory effect (16). Also, effect of quercetin on triple transgenic mice of Alzheimer’s disease model has been studied and protective effects on performance, emotional and cognitive behavior have been confirmed on brain cells (30). In another study conducted in 2015, pharmacokinetics and mechanism of neuroprotective effect of quercetin was studied in mice representing strong protective effects of this compound on the brain cells (31).

## Conclusion

This study was conducted for the first time on *Leutea avicennia*, and showed this plant is rich of phenolic compounds that exhibited appropriate antioxidant and anti- acetylcholinesterase properties. Caffeic acid is one of the substances which showed potent activity in both assays, therefore methanol fraction of *L.avicennia* and their components probably could be used in treatment of neurodegenerative disease, so *in-vivo* animal model is worthwhile and recommended. 
